# Randomised control trials using short messaging services (SMS) in hypertension treatment – convenient telemedicine interventions

**DOI:** 10.1080/02813432.2022.2159636

**Published:** 2023-01-06

**Authors:** Susanna Calling, Hans Thulesius

**Affiliations:** a Center for Primary Health Care Research, Department of Clinical Sciences Malmö, Lund University, Region Skåne, Sweden; b Department of Clinical Sciences Malmö, Family Medicine, Lund University, Lund, Sweden; c Research and Development Centre, Region Kronoberg, Växjö, Sweden; d Department of Medicine and Optometry, Linnaeus University, Kalmar, Sweden

A 2003 SJPHC opinion paper on Frontline Ideas titled ‘Joining the mobile revolution’ [1] promoted the use of mobile phones’ short messaging services (SMS) in general practice. The focus was SMS reminders in preventive care and for appointment booking purposes. The author predicted the future well. Yet, finding the optimal use of SMS to improve health is a work in progress according to the ubiquitous scientific literature on the subject [2,3].

The SJPHC paper of the year 2019 – ‘Having to learn this so late in our lives…’ Swedish elderly patients’ beliefs, experiences, attitudes, and expectations of e-health in primary health care’ [4] shows that ease of use is crucial. This indeed goes well with SMS as a telemedicine tool. Hence, since elderly’s health issues make up a big and growing part of health care we should probably be using SMS more to improve care for elderly in general and for hypertension management in particular according to evidence [5]. Also, since high blood pressure is a lead cause of mortality worldwide [6] and hypertension is under treated according to the ‘rule of halves’ or in Sweden ‘rule of thirds’ – one half or one third of the population with chronic conditions such as hypertension are not known, half of those known are not treated and half of those treated are not controlled [7,8] – the use of SMS to manage hypertension is probably not utilised to its full potential.

To improve antihypertensive drug treatment, SMS was tested in the Check and Support study, a cluster randomised control trial (RCT) of 111 patients 30–75 years old with newly diagnosed hypertension in Finland [9]. The intervention consisted of personalised SMS-messages and checklist support for treatment initiation which was delivered to 57 participants whilst 54 participants got usual care. Every intervention participant received at least 43 SMS-messages over 12 months. No significant differences in lowered blood pressure levels were seen − 23 mm Hg office systolic blood pressure reduction in the intervention group and 21 mm reduction in the control group (*p* = .61). The proportion of participants who reached SBP targets in the intervention and control groups were 28% and 31%, respectively (*p* = .52). Medication changes, number of antihypertensives and health care utilisation were also similar in both groups. Yet, results of blood pressure measurements presented in a box-plot-diagram in the paper showed a possible positive effect of the intervention regarding home SBP. Also, checklist and text message support were considered useful and important by most participants.

A Swedish six month RCT pilot study of SMS-messages for treatment of hypertension included 60 primary care patients aged 40–80 years with hypertension [10]. Four lifestyle-promoting text messages were sent every week for six months to 30 patients (=104 messages per patient). The control group of 30 patients received usual care. The baseline and follow-up visits for all 60 patients included measurements of blood pressure, anthropometrics, blood tests and a self-reported questionnaire. The feasibility outcomes for the pilot study (recruitment, dropout and eligibility) were fulfilled. A favourable yet non-significant trend of improvements in all 11 predefined cardiovascular risk factors were seen. Moreover, the intervention patients reported a high acceptance of the SMS intervention with 38% stating that the SMS made them more physically active and 24% reported that they had changed to healthier dietary habits. An ongoing full scale study titled PUSHME (Primary care Usage of Health promoting Messages), based on the pilot study, will complete the recruitment of 400 patients in December 2022 [clinicaltrials.gov: NCT03442257].

A main difference between the interventions were the number of messages delivered to the patients which were higher in the Swedish study. The Finnish patients were younger and newly diagnosed with hypertension whilst the Swedish patients had hypertension since before and thus a much lower baseline blood pressure value than the Finnish patients. These two recent Nordic RCTs of SMS to improve outcome in hypertension treatment in primary care did not give clear evidence for SMS being a useful tool even if especially the Swedish study showed promising results. Why? We believe that this is probably explained by underpowered design preventing the detection of small benefits of the interventions and the Swedish study indeed being a pilot intervention.

Another explanation may be a low motivation to lifestyle modifications in patients without manifest cardiovascular disease which makes primary prevention a difficult challenge. The Australian RCT TEXTME studied lifestyle-focused SMS in patients with previous coronary heart disease and found significant improvement in several cardiovascular risk factors including reduced low density lipoprotein cholesterol and systolic blood pressure [11]. However, the following RCT TEXTME2 that studied lifestyle SMS for primary prevention found no significant reduction of uncontrolled cardiovascular risk factors [12]. Yet, the participants in the intervention group improved cardiovascular risk factor control overall during the study period and were less likely to be physically inactive [12].

As most conclusions we also conclude that more evidence is needed in this field of support for hypertension treatment by using the SMS text function of the ever-present personal cell phone as a convenient digital telemedicine tool.
